# Case Report: Uniportal robot-assisted thoracoscopic double-sleeve lobectomy after neoadjuvant immunotherapy

**DOI:** 10.3389/fsurg.2024.1360125

**Published:** 2024-02-20

**Authors:** Ziyao Fang, Chang Li, Mugurel L. Bosinceanu, Cheng Ding, Jun Zhao, Diego Gonzalez-Rivas

**Affiliations:** ^1^ Department of Thoracic Surgery, The First Affiliated Hospital of Soochow University, Suzhou, China; ^2^ Research Center of Thoracic Surgery, The First Affiliated Hospital of Soochow University, Suzhou, China; ^3^ Department of Thoracic Surgery, Policlinico di Monza, Oncology Hospital Monza, Bucharest, Romania; ^4^ Department of Thoracic Surgery and Minimally Invasive Thoracic Surgery Unit (UCTMI), Coruña University Hospital, Coruña, Spain

**Keywords:** sleeve resection, robot-assisted thoracoscopic surgery (RATS), uniportal video-assisted thoracoscopy (uniportal VATS), lung cancer, minimally invasive and robotic surgery

## Abstract

Minimally invasive thoracic surgery, including video-assisted thoracoscopic surgery and robot-assisted thoracoscopic surgery, has been proven to have an advantage over open thoracotomy with less pain, fewer postoperative complications, faster discharge, and better tolerance among elderly patients. We introduce a uniportal robot-assisted thoracoscopic double-sleeve lobectomy performed on a patient following neoadjuvant immunotherapy. Specialized instruments like customized trocars with a reduced diameter, bulldog clamps, and double-needle sutures were utilized to facilitate the maneuverability through the single incision. This technique integrates the merits of multiport robot-assisted thoracic surgery with uniportal video-assisted thoracoscopic surgery.

## Introduction

Sleeve lobectomy has been proved to provide a better quality of life compared with pneumonectomy, with reduced morbidity and mortality rates specifically for centrally located tumors (1–3). Double-sleeve resection is defined as a surgery to remove one lobe with a part of the main bronchus and a part of the pulmonary artery (PA). Afterward, anastomosis is performed between both the ends of the bronchus and the pulmonary artery (4). Minimally invasive thoracic surgery encompasses video-assisted thoracoscopic surgery (VATS) and robot-assisted thoracoscopic surgery (RATS). These approaches offer advantages over open thoracotomy, including reduced pain, decreased incidence of postoperative complications, expedited discharge, and improved tolerance among elderly patients (5, 6). Over the past decade, minimally invasive thoracic surgery has developed from utilizing three or four ports to a single incision, which is known as uniportal video-assisted thoracoscopic surgery (U-VATS) (7, 8). Meanwhile, the adoption of RATS has gradually increased around the world, demonstrating technical advantages including tremor filtration, improved maneuverability, and three-dimensional view (9, 10). To integrate the merits of U-VATS and RATS, Gonzalez-Rivas et al. analyzed the Da Vinci Surgical System Xi and performed the first uniportal robot-assisted thoracic surgery (U-RATS) lobectomy in September 2021. Since then, U-RATS has been applied to various surgical procedures, including sleeve resections (11). We present a case of a patient who underwent U-RATS double-sleeve lobectomy of the left upper lobe.

## Case presentation

**Figure 1:**
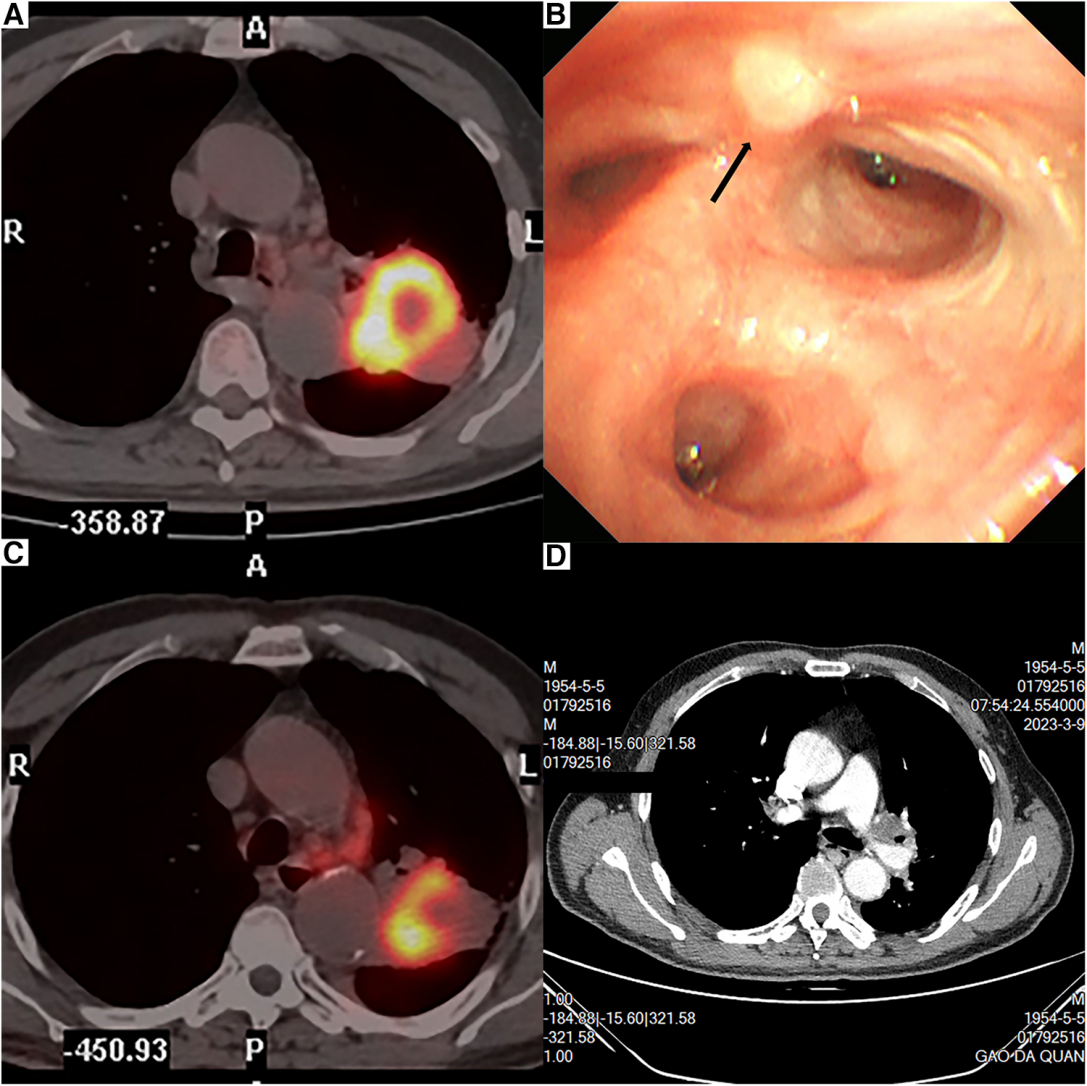
PET revealed a 61.7-mm × 43.9-mm tumor near the left hilum (**A**). PET examination after immunotherapy revealed that the tumor was 45 mm × 44 mm (**B**). Bronchoscopy result (**C**). Contrast-enhanced CT revealed that the tumor invaded the left upper bronchus and the left upper artery (**D**).

The patient was a 68-year-old man with no smoking history. A computed tomography (CT) scan revealed a mass, 61.7 mm × 43.9 mm in size, near the left hilum at the left upper lobe. A ^18^F-fluorodeoxyglucose (FDG)-positron emission tomography (PET) showed increased accumulation of FDG in the mass (standardized uptake value: 15.08) and in the enlarged lymph node at the left hilum (standardized uptake value: 5.78) (Figure 1A). The bronchoscopy confirmed the mass at the origin of the light upper lobe bronchus (Figure 1B). Pulmonary squamous cell carcinoma was diagnosed by the bronchoscopic biopsy. Thus, clinical T3N2M0 pulmonary squamous cell carcinoma was diagnosed according to the eighth edition of the Tumor, Node, Metastasis (TNM) classification for lung cancer (12). The patient was scheduled to receive neoadjuvant therapy consisting of monthly administrations of paclitaxel (210 mg), carboplatin (0.5 g), and tislelizumab (200 mg) for a duration of 3 months. However, the patient complained of chest pain after pumping of 17 ml paclitaxel intravenously. The chemotherapy was discontinued and his chest pain was relieved. Consequently, the patient received tislelizumab only as immunotherapy for three cycles. PET examination after completing three cycles of immunotherapy revealed the mass was 45 mm × 44 mm in size and the standardized uptake value was 12.31 (Figure 1C). The tumor size decreased and was evaluated as a stable disease (SD) after immunotherapy. Preoperative contrast-enhanced CT revealed tumor infiltration of the left upper bronchus and PA (Figure 1D). The patient was proposed for a U-RATS double-sleeve resection of the left upper lobe.

## Surgical technique

The patient was placed in lateral decubitus on the right side with double-lumen endotracheal intubation. A 4-cm incision was made in the sixth intercostal space at the middle axillary line and a wound protector was placed. The robot was docked on the dorsal side of the patient with Arm No.1, Arm No.2 for instruments, Arm No.3 for camera, and Arm No.4 as canceled. The instruments for VATS and open surgery were prepared in case of need. The assistant surgeon manipulated the aspirator and delivered the sutures in need through the same incision.

The oblique fissure was initially opened by a cautery hook and endoscopic staplers to expose the PA. Then, the interlobular lymph nodes (station 11) were exposed adjacent to the superior segmental artery (A^6^). We divided the inferior pulmonary ligament and removed lymph nodes stations 8 and 9. The upper lobe was anteriorly retracted to expose the posterior hilar tissue, and the left main bronchus was dissociated to remove lymph nodes stations 7 and 10. Afterward, the upper lobe was posteriorly retracted to expose the anterior mediastinum. We dissected the anterior mediastinum to expose the superior pulmonary vein and the left main PA. During the process, lymph node station 5 was removed.

**Figure 2:**
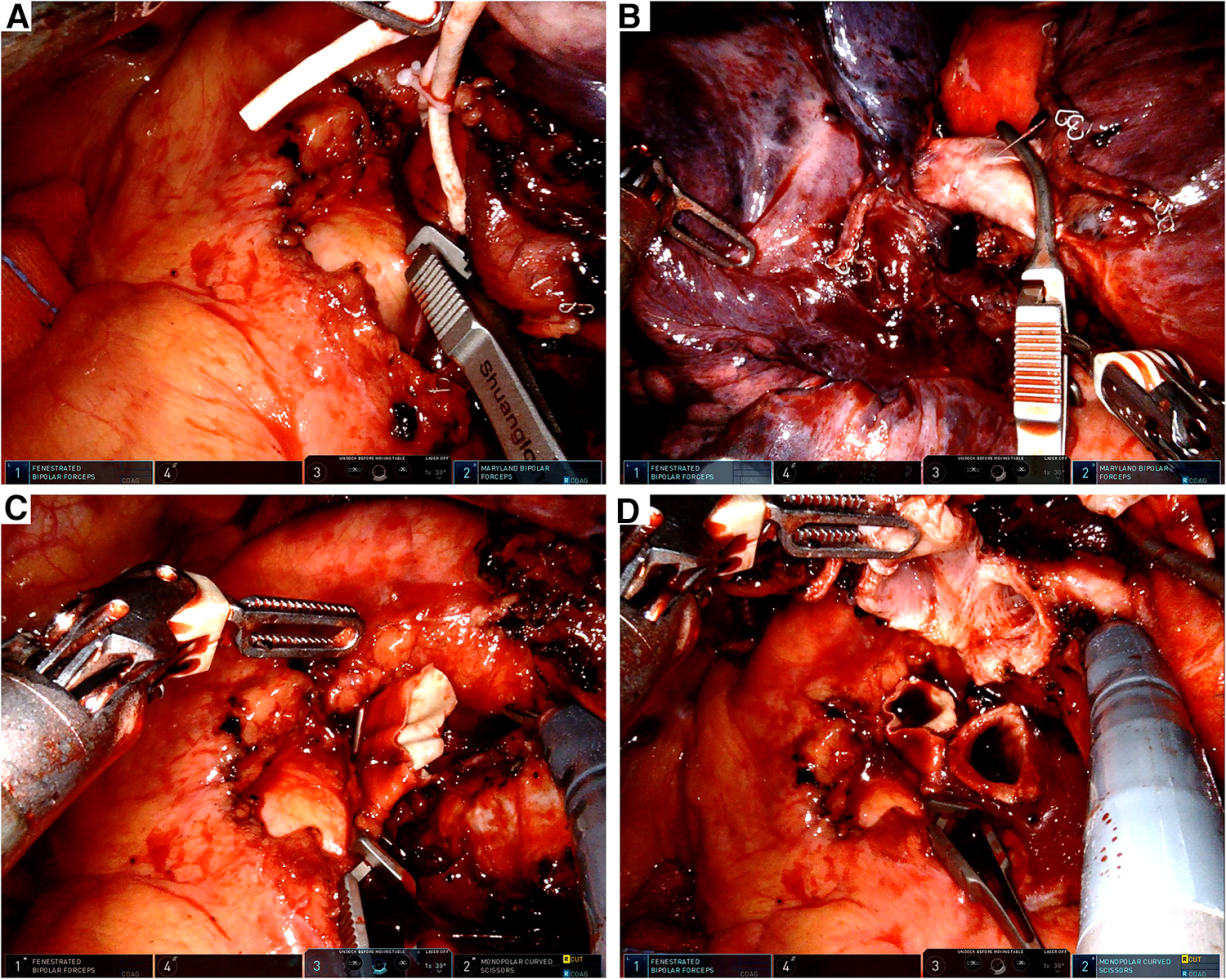
Intraoperative view. (**A**) Left main PA was blocked by a bulldog clamp. (**B**) Distal PA was blocked by a bulldog clamp. (**C**) Left main PA was transected by the robotic scissor. (**D**) Left main bronchus was cut open by the robotic scissor.

A bulldog clamp was placed to block off the proximal left main PA (Figure 2A) and another bulldog clamp was placed at the origin of A^6^ to block off the distal PA (Figure 2B). We cut open the blocked PA between the two bulldog clamps (Figure 2C). Subsequently, the left main bronchus was cut open proximal to the origin of the left lower bronchus (Figure 2D). The left upper lobe was put into the specimen bag and pulled out through the incision for the frozen section. The frozen sections of resection margins were negative.

**Figure 3:**
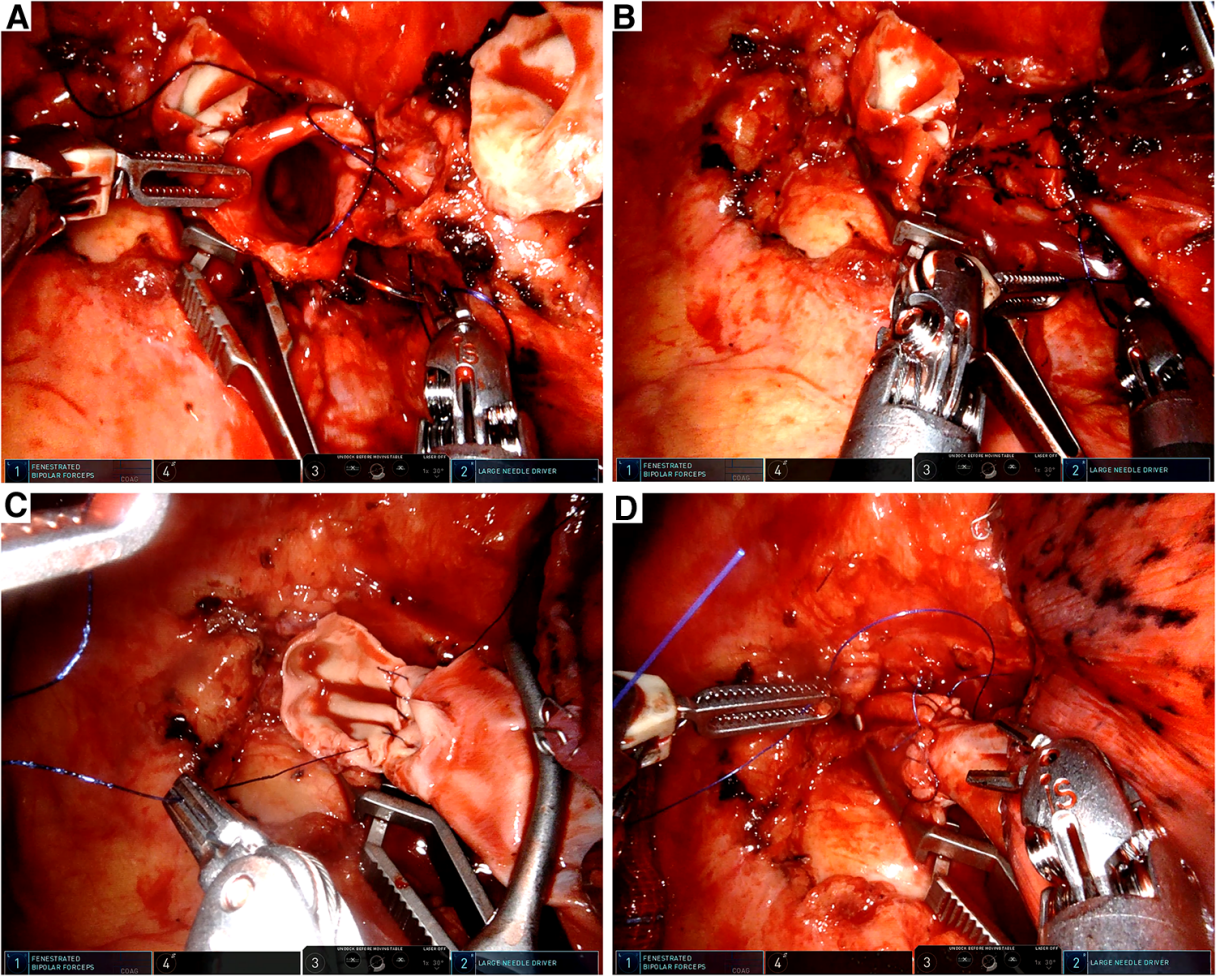
Intraoperative view. (**A**) Anastomosis of the left main bronchus and the left lower bronchus. (**B**) Tracheal anastomosis was completed with a running suture. (**C**) Vascular reconstruction of the left main PA and the left lower PA. (**D**) Vascular reconstruction was completed by a running suture and fortified by a mattress suture.

**Figure 4:**
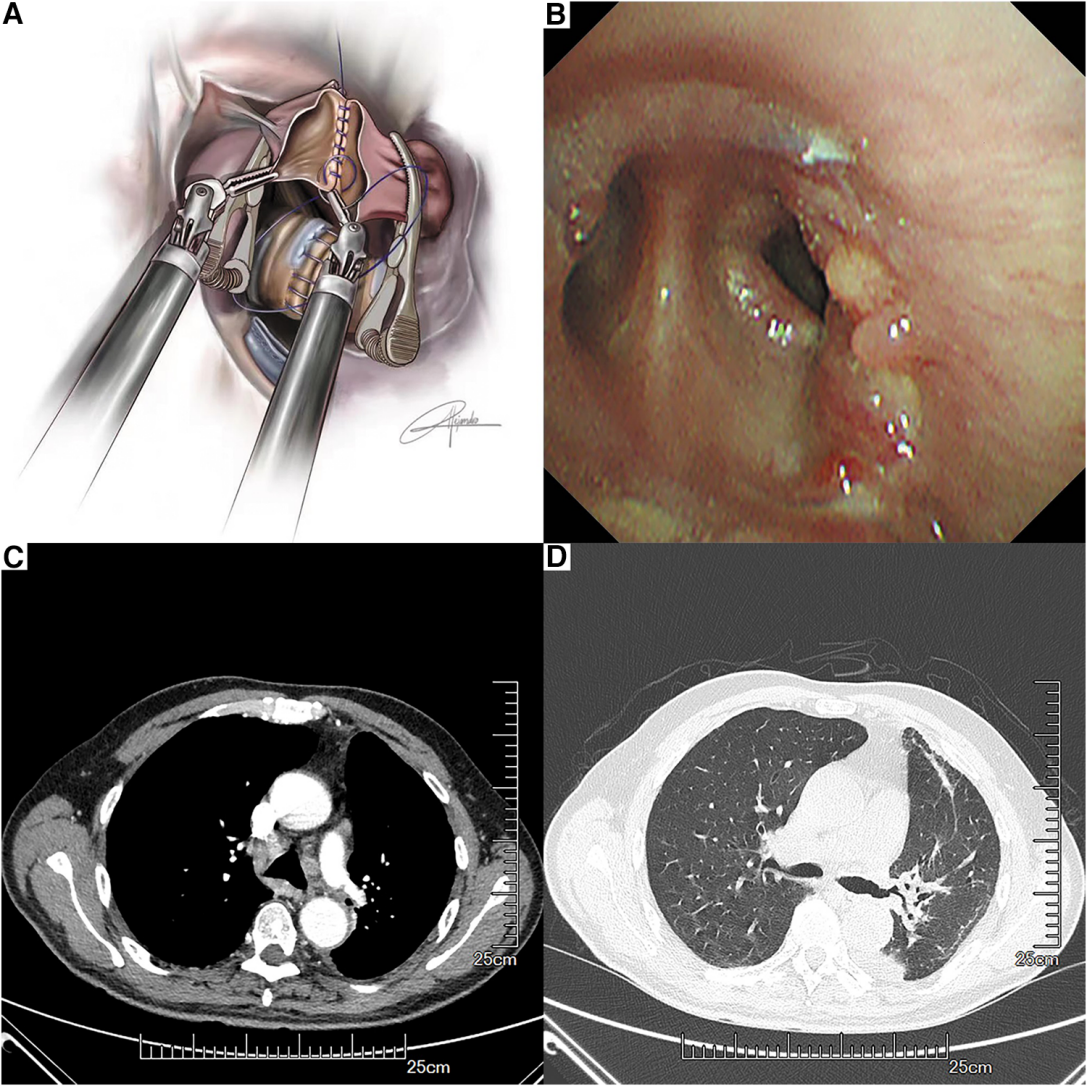
Anastomosis of the left pulmonary artery and the left lower pulmonary artery with a running suture by using bulldog clamps (**A**). Bronchoscopy findings at the anastomotic site on the 50th postoperative day (**B**). Recent contrast-enhanced CT (**C,D**).

First, the anastomosis of the bronchus was performed by an absorbable barbed double-needle 3-0 suture (Figure 3A). The running suture started from the posterior wall of the left lower bronchus (in-out) to the posterior wall of the left main bronchus (out-in). The membranous part was sutured first and then the cartilaginous part was sutured in a circumferential manner (Figure 3B). Water test was conducted and no air leak was observed. Then we conducted the vascular anastomosis using a double-needle 5-0 prolene suture in a continuous style. The artery angioplasty also began at the posterior wall of the interlobar artery and the left main PA (Figure 3C, Figure 4A). After the artery angioplasty, mild exudation was observed at the superior portion of the anastomosis. We employed another single-needle 5-0 prolene suture to fortify the anastomosis by mattress suture (Figure 3D). During the suturing, the artery lumen was irrigated by heparin solution regularly. Finally, the bulldog clamps were removed and we wrapped the anastomosis with gelatin sponge and biogel.

## Outcome

The operation time was about 320 min and the intraoperative blood loss was about 200 ml. The patient was extubated in the operation room and was transferred to the intensive care unit for one day. The chest x-ray on the first postoperative day suggested that the lung was well ventilated. The total chest tube drainage was about 600 ml for 4 days and the chest tube was removed on the postoperative day 4. The patient was discharged on postoperative day 5 without any complications. The lesion on the gross sample exhibited a diameter of 4 cm, and the margin of the bronchus and the vessel was negative. The lymphatic metastasis was positive at station 12, while stations 5–11 were negative. The postoperative pathological diagnosis of the patient revealed stage IIB squamous cell carcinoma, T2N1M0. Bronchoscopy examination on the 50th postoperative day suggested satisfactory healing of the anastomosis (Figure 4B). A recent contrast-enhanced CT (9 months after the surgery) did not reveal any apparent complications or recurrence (Figures 4C,D). A figure showcasing the timeline of the patient is presented in Supplementary Figure S1.

## Discussion

With the development of robotic technologies, RATS has been increasingly accepted for lung cancer treatment. RATS combines the advantages of VATS with precision of movements, trembling filtration, and three-dimensional surgical view (10). However, RATS still requires four or five ports, while VATS requires only one incision since the first U-VATS was completed in 2011 (7). Compared with traditional VATS, the uniport technique possesses virtues of less perioperative pain, shorter postoperative hospital stay, and a sagittal view for the surgeon (13–15). To converge the advantages of RATS and U-VATS, Gonzalez-Rivas and Ismail managed to perform several uniport thoracic surgery-based cadaver experiments by the da Vinci SP system in 2018. However, the SP system is not widely applied because of the higher financial cost and lack of integrated robotic staplers (16). Gonzalez-Rivas et al. managed to perform the first U-RATS lobectomy in September 2021 (11). Since then, U-RATS has been gradually accepted globally. In 2022, Ning et al. performed a right upper lobe and carinal sleeve resection through U-RATS (17). Paradela et al. compared the early outcomes of U-RATS and U-VATS and confirmed the safety, feasibility, and efficacy of U-RATS in 2022 (15). In 2023, Manolache et al. conducted a multicenter study to compare the outcomes of U-RATS and standard RATS. The study suggests that U-RATS and standard RATS exhibit comparable rates of negative resection margins, lymph node resections, and complications. By contrast, U-RATS offers several advantages over standard RATS including reduced conversion rate to thoracotomy and shorter operative time and duration of postoperative hospital stay (18).

We adopted the same three-arm U-RATS technology as Gonzalez-Rivas et al. and Ning et al. in the presented case. Differences between U-RATS and traditional or hybrid RATS in surgical techniques should be mentioned. Robotic staplers were applied so that the main surgeon could have a better control over the surgery. The incision was made in the sixth intercostal space, which was lower than hybrid RATS, to create a good angle for robotic stapler movement. According to Gonzalez's experience in U-VATS double-sleeve resections, we used two bulldog clamps for both the main PA and the distal artery to allow for more space through the incision (19). The transection of the artery preceded that of the bronchus while the anastomosis of the bronchus preceded that of the artery to minimize the tension on artery anastomosis. Both the PA and the bronchus were reconstructed by running suture. Compared with interrupted suture, running suture reduces the anastomosis time and avoids knot tangling. There has been a controversy on whether to wrap or not wrap the anastomosis by tissue flaps (20). Campisi et al. conducted a retrospective multicenter analysis in 2021 to compare the postoperative outcomes between patients with or without bronchial wrapping and confirmed that sleeve resections may be performed safely without bronchial wrapping (21). Given the challenges to obtain suitable tissue flaps, we employed biomaterials to protect the anastomosis.

Concerns about an increased risk of anastomosis complications after neoadjuvant therapy have been raised since preoperative induction treatments may lead to impaired bronchial blood supply and fibrotic alterations (22, 23). Koryllos et al. carried out prospective research to compare the bronchial healing status in patients with or without neoadjuvant therapy. The result indicates that radiotherapy or chemoradiotherapy could increase the risk of pulmonary complications (24). However, neither chemoimmunotherapy nor chemotherapy has been reported to pose a negative effect on postoperative recovery of sleeve resections (25, 26). In the presented case, the patient received neoadjuvant immunotherapy alone and manifested a smooth postoperative recovery. No obvious fibrotic alterations or impaired blood supply were observed during the surgery. This indicated to us that uniportal RATS double-sleeve resection following neoadjuvant immunotherapy is feasible but should be performed by experienced surgeons who are familiar with the technique.

## Conclusion

Herein, we have presented a case of U-RATS double-sleeve resection after neoadjuvant immunotherapy. U-RATS is a promising technique that combines the advantages of RATS and U-VATS. This technique should be taken into account if a patient is scheduled to receive complex thoracic surgery.

## Data Availability

The original contributions presented in the study are included in the article/**Supplementary Material**, further inquiries can be directed to the corresponding authors.

## Supplementary material

Supplementary Figure S1 Timeline of the course of the disease.
