# Supplementary figures and images for: Case Report: Uniportal robot-assisted thoracoscopic double-sleeve lobectomy after neoadjuvant immunotherapy
Source: Front Surg. 2024 Feb 20;11:1360125. doi: 10.3389/fsurg.2024.1360125 (PMC10912180; doi:10.3389/fsurg.2024.1360125)

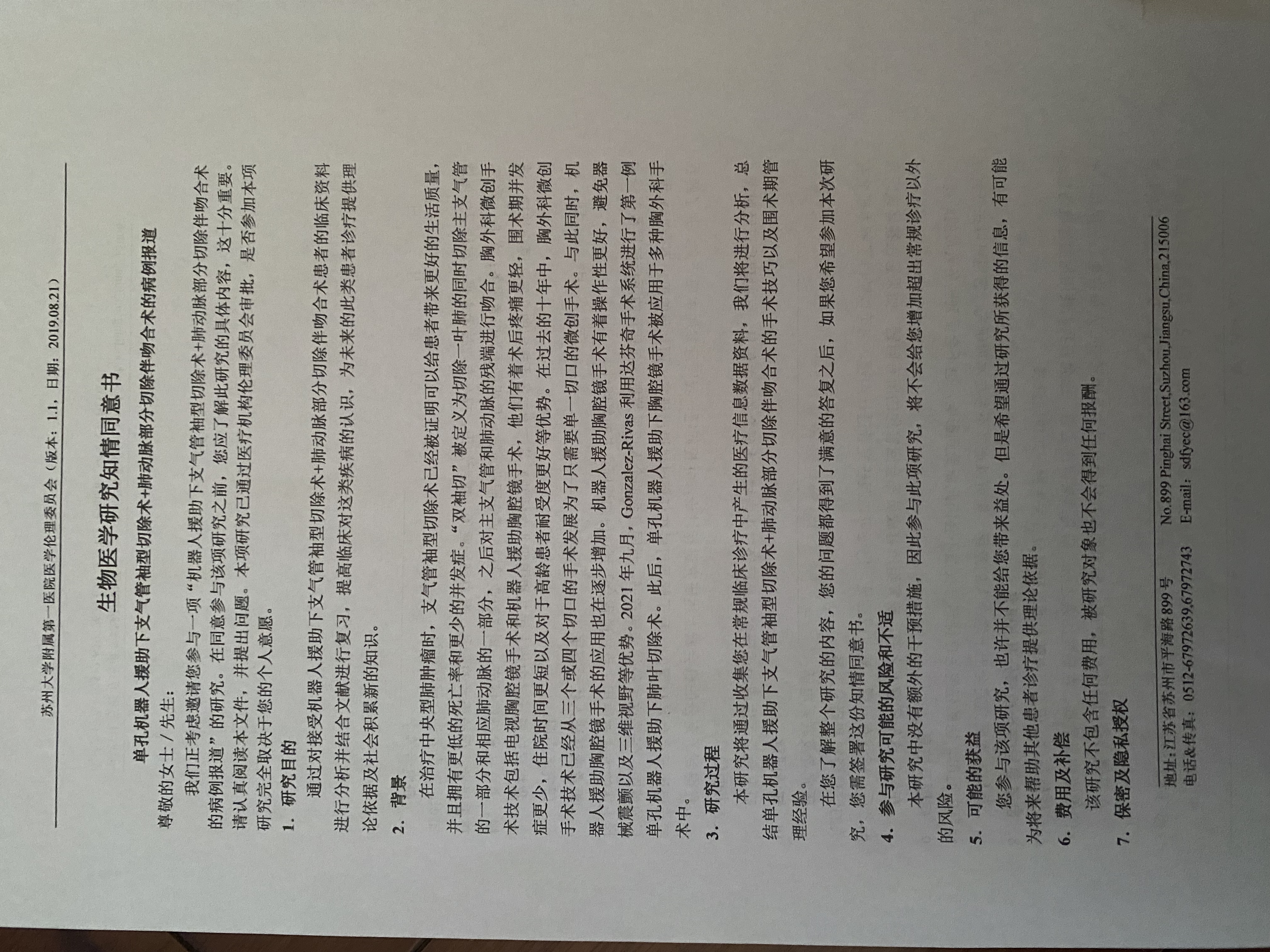

Supplement: Supplementary Figure S1 — Timeline of the course of the disease. [file Image1.jpeg]

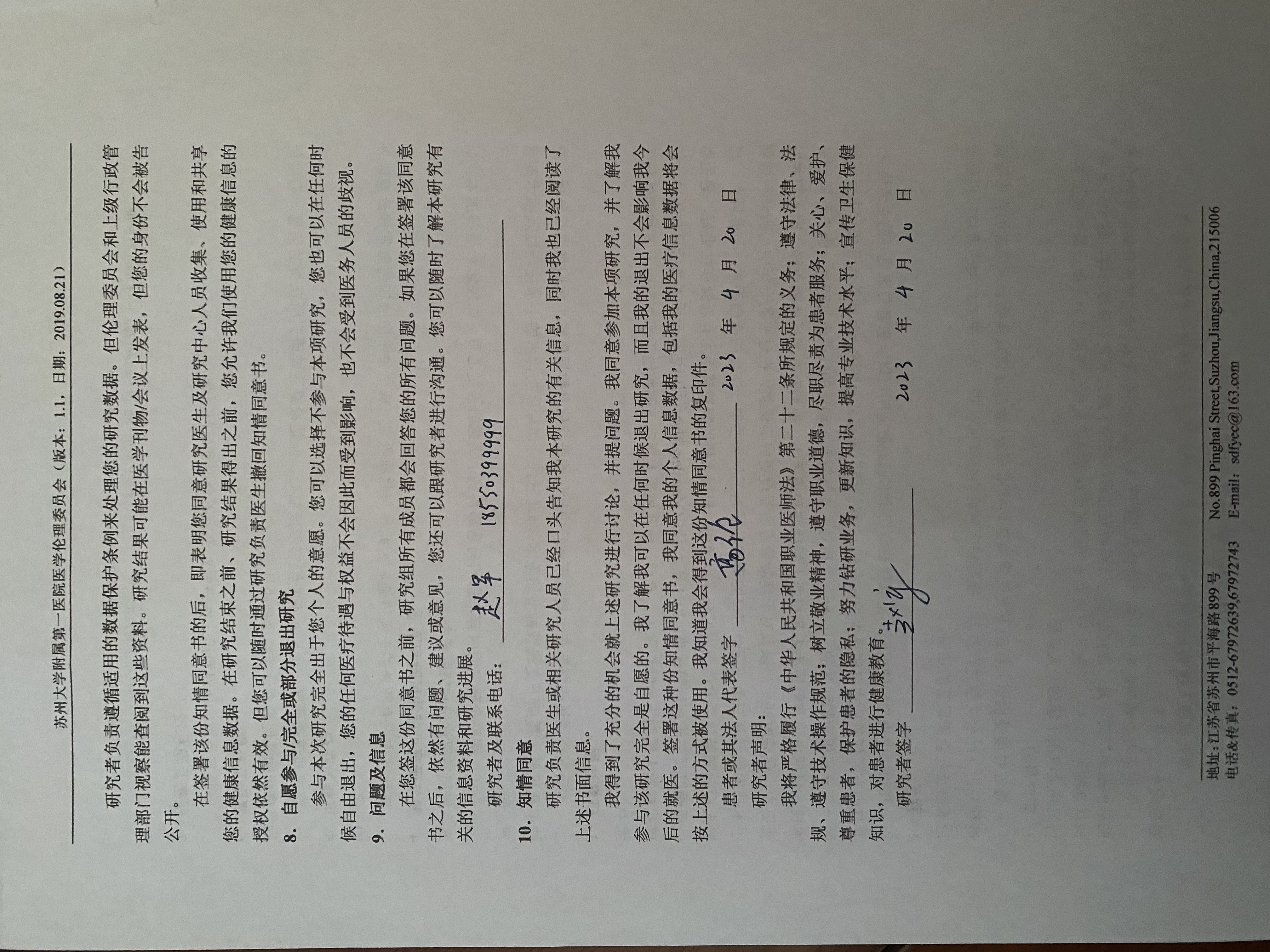

Supplement: Supplementary file 2 [file Image2.jpeg]

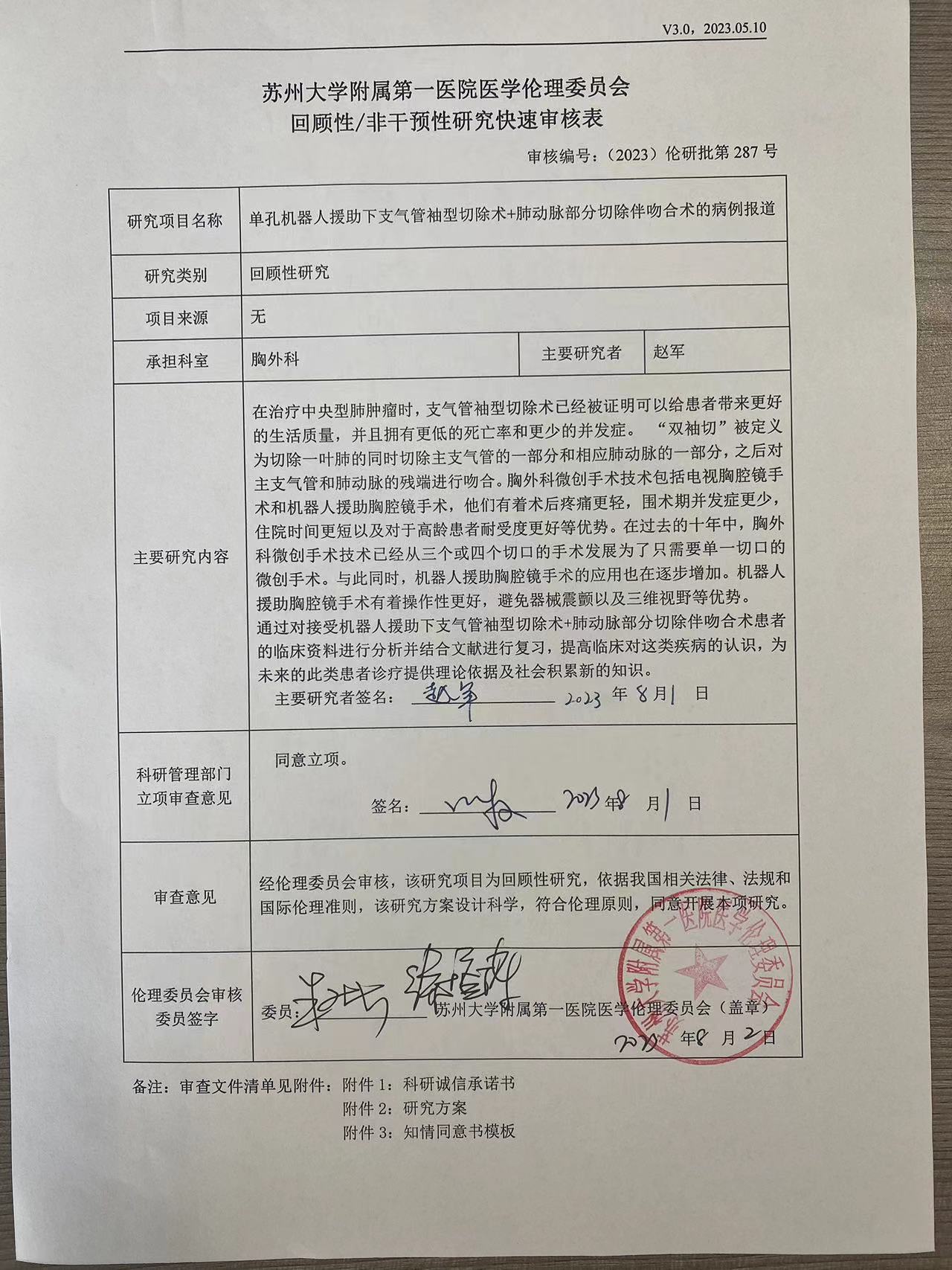

Supplement: Supplementary file 3 [file Image3.jpeg]

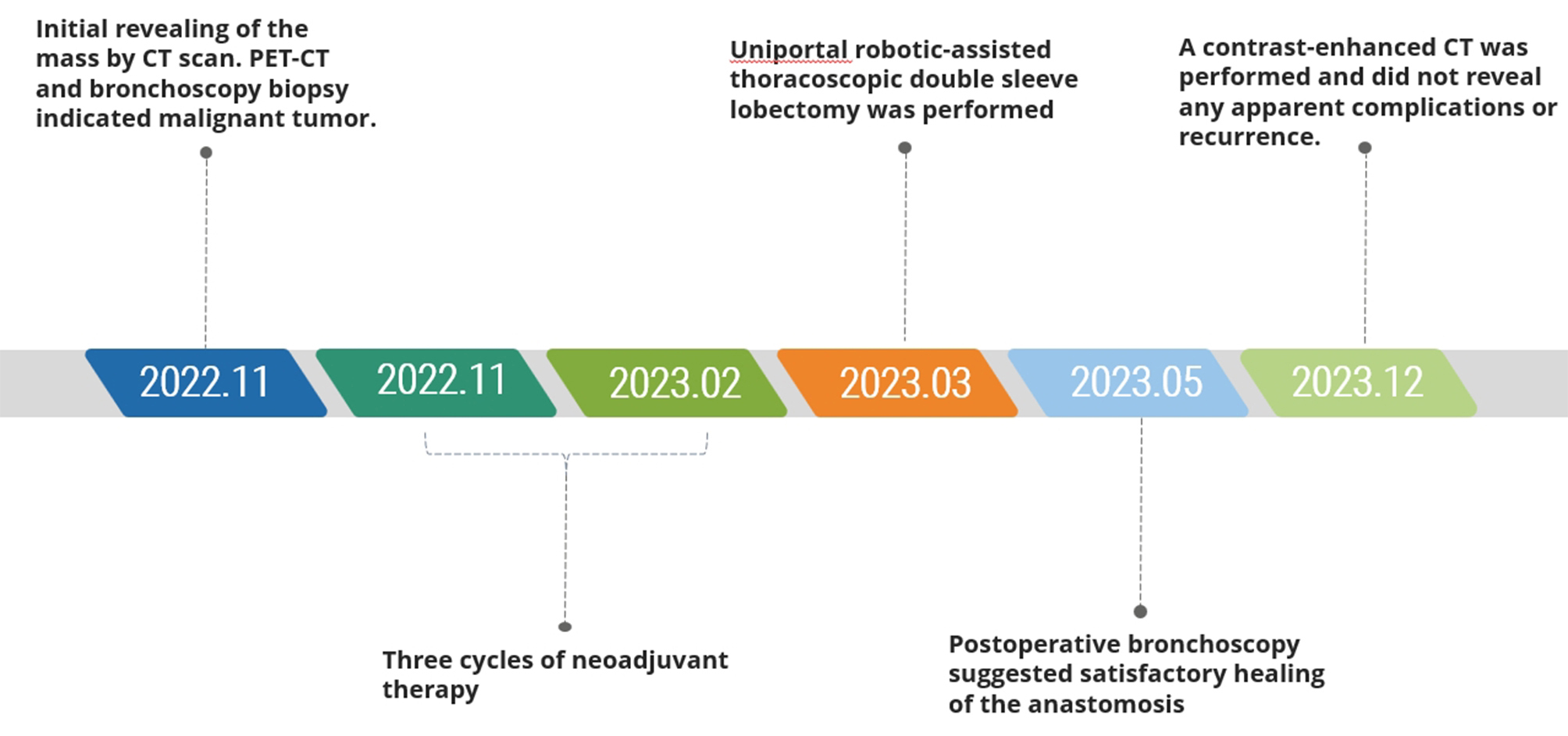

Supplement: Supplementary file 4 [file Image4.jpeg]
